# GATA2 Deficiency in Adult Life Is Characterized by Phenotypic Diversity and Delayed Diagnosis

**DOI:** 10.3389/fimmu.2022.886117

**Published:** 2022-05-06

**Authors:** Oded Shamriz, Naseem Zahalka, Amos J. Simon, Atar Lev, Ortal Barel, Nofar Mor, Yuval Tal, Michael J. Segel, Raz Somech, Hagith Yonath, Ori Toker

**Affiliations:** ^1^ Allergy and Clinical Immunology Unit, Department of Medicine, Hadassah Medical Organization, Faculty of Medicine, Hebrew University of Jerusalem, Jerusalem, Israel; ^2^ The Lautenberg Center for Immunology and Cancer Research, Institute of Medical Research Israel-Canada, Faculty of Medicine, Hebrew University of Jerusalem, Jerusalem, Israel; ^3^ Internal Medicine A, Sheba Medical Center, Ramat Gan, Israel; ^4^ Sheba Cancer Research Center and Institute of Hematology, Sheba Medical Center, Ramat Gan, Israel; ^5^ Pediatric Department A and the Immunology Service, Jeffrey Modell Foundation Center, Edmond and Lily Safra Children’s Hospital, Tel-Hashomer Medical Center, Affiliated to the Sackler Faculty of Medicine, Tel Aviv University, Tel Aviv, Israel; ^6^ The Jeffrey Modell Foundation Israeli Network for Primary Immunodeficiency, New York, NY, United States; ^7^ The Genomic Unit, Sheba Cancer Research Center, Sheba Medical Center, Ramat Gan, Israel; ^8^ Sheba Medical Center, Wohl Institute of Translational Medicine, Ramat Gan, Israel; ^9^ Sackler Faculty of Medicine, Tel Aviv University, Tel Aviv, Israel; ^10^ Department of Pulmonology, Sheba Medical Center, Ramat Gan, Israel; ^11^ Danek Gertner Institute of Human Genetics, Sheba Medical Center, Ramat Gan, Israel; ^12^ Allergy and Clinical Immunology Unit, Shaare Zedek Medical Center, Faculty of Medicine, Hebrew University of Jerusalem, Jerusalem, Israel

**Keywords:** GATA2, exome, primary immune deficiency (PID), hematopoietic, diagnosis

## Abstract

The transcription factor GATA2 plays a key role in the survival and self-renewal of hematopoietic stem and progenitor cells. Autosomal dominant variants in *GATA2* cause a broad spectrum of heterogeneous phenotypes. Here, we present our experience with GATA2 deficiency in a retrospective multicenter analysis of computerized medical records of adult patients (age ≥18 years) treated between 2018 and 2022 at Shaare Zedek Medical Center in Jerusalem and Sheba Tel-Hashomer Medical Center in Ramat Gan, Israel. Two male and two female patients with GATA2 deficiency were identified. Three of the patients presented with symptoms in adult life and all patients were diagnosed as adults. Age at presentation was 10.5-36 years and age at diagnosis 24-47 years. Diagnosis was delayed in all patients by 1-24.5 years. The phenotypic diversity was notable. Patients presented with myelodysplastic syndrome (n=2), pulmonary alveolar proteinosis (n=1), and recurrent viral (n=1), bacterial (n=3), and mycobacterial (n=1) infections. Bone marrow biopsy revealed cytogenetic abnormalities in one patient (monosomy 7). Patients were diagnosed by exome sequencing (n=3) and Sanger sequencing of the coding exons in *GATA2* (n=1). Novel heterozygous *GATA2* variants (c.177C>A, p.Y59* and c.610dup, p.R204Pfs*78) were identified in two patients. Immune workup revealed B cell lymphopenia and monocytopenia in all tested patients. One patient died from overwhelming sepsis despite all patients being treated with antibiotics and anti-mycobacterials. Our cohort highlights the phenotypic diversity, late presentation, and delayed diagnosis of GATA2 deficiency. Increased awareness of this primary immune deficiency presenting in adult life is needed and should involve a high index of suspicion.

## Introduction

GATA2 is a transcription factor that plays a key role in the survival and self-renewal of hematopoietic stem and progenitor cells (HSPCs). *Via* its two zinc fingers (ZF), GATA2 regulates the expression of different genes involved in the maintenance of HSPCs by both protein-protein and protein-DNA interactions (1).

Autosomal dominant-inherited variants of *GATA2* that induce protein loss-of-function or deficiency were previously reported to present with heterogeneous phenotypes and a broad spectrum of clinical manifestations (1, 2). Patients are characterized by increased susceptibility to bacterial, mycobacterial, viral, and invasive fungal infections. In late childhood and early adulthood, GATA2 deficiency patients commonly develop monocytopenia with mycobacterial disease (MonoMAC) (3). In addition, patients are characterized by natural killer (NK) and B cell cytopenia, defective antiviral and proinflammatory responses of the innate system and impaired type I interferon production with a defective T-cell response (4). Viral infections include human papillomavirus (HPV), molluscum contagiosum, herpes simplex virus, Epstein-Barr virus, and cytomegalovirus (CMV) (5).

Myelodysplastic syndrome (MDS) appears with cytogenetic abnormalities in the bone marrow, such as monosomy 7, as well as increased susceptibility to hematological malignancies, mainly acute myeloid leukemia (1, 2, 6). Other clinical presentations may include chronic neutropenia, aplastic anemia, lymphedema, pulmonary alveolar proteinosis (PAP), and hearing loss (1, 2, 6, 7).

Diagnosis of GATA2 deficiency is often delayed, and many affected patients are diagnosed with hematological malignancies within the first four decades of life (8). Studies focusing on GATA2 deficiency within the adult population are scarce. Jørgensen et al. reported of 11 adult GATA2-deficient patients. Adult patients were diagnosed by hematologists, infectious disease specialist and family genetic screening (9) West et al. investigated a large cohort of adult patients, some with secondary occurring variants in ASXL1 and STAG2 (10).

Here, we describe our experience with GATA2 deficiency in adult patients to shed light on the immune and hematological features.

## Methods

### Study Design and Patients

In this retrospective study, we analyzed adult patients (age ≥18 years) with GATA2 deficiency, who were admitted during the period of 2018-2022 to Shaare Zedek Medical Center in Jerusalem and Sheba Medical Center in Ramat-Gan, Israel. Data was retrieved from the patients’ computerized medical records and the diagnosis was obtained by immune and genetic workups.

### Genetic Analysis

Exome sequencing (ES) was performed using the Twist Human Core Exome Plus Kit (Twist Bioscience, San Francisco, CA, USA) on a NovaSeq 6000 sequencing machine (Illumina, San Diego, CA, USA). For each sample, paired end reads (2 × 100 bp) were obtained and processed. The Dragen Bio-IT Platform (version 3.8; Illumina) was used to align reads to the human reference genome (hg38) based on the Smith-Waterman algorithm (11) and to call variants based on the GATK variant caller (version 3.7) (12). Additional variants were called with Freebayes (version 1.2.0) (13). Variants were annotated using KGG-Seq (version 1.2) (14). Further annotation and filtration steps were performed using in-house scripts and various additional datasets, both public [e.g., HGMD (Stenson), ClinVar (Landrum), gnomAD (Karczewski)] and from our own database of variants in previous sequencing (~6000 exomes and ~100 genomes drawn from the Israeli population).

### Immune Workup

Cell surface markers of peripheral blood mononuclear cells were measured by flow cytometry (NAVIOS; Beckman Coulter) with immunofluorescent staining using anti-CD3, anti-CD4, anti CD8, anti-CD19, anti-CD16, and anti-CD56 antibodies (Beckman Coulter). T-cell receptor (TCR) v-β expression was determined as directed by the manufacturer (Beta Mark TCR v-β Repertoire Kit; Beckman Coulter). Reference range for lymphocyte subsets in adults were taken from Apoil et al. (15)

### Ethical Review of the Study

The study was approved by the Institutional Review Board (IRB) of Sheba Tel-Hashomer Medical Center (8842-11-SMC). A waiver for conducting this study was received from the IRB of Shaare Zedek Medical Center. A waiver for participants consent was gained by IRBs of both centers.

## Results

### Clinical Characteristics of the Patients

Four adult patients (P1-P4) with GATA2 deficiency were identified. The clinical characteristics of the patients are detailed in **Table 1**. Two patients were males. Consanguinity and family history of GATA2 deficiency were noted in none of the patients. P2-P4 presented with symptoms in adult life, but all patients were diagnosed as adults. Age at presentation was 10.5-36 years and age at diagnosis 24-47 years. Diagnosis was delayed in all patients with a period from presentation to diagnosis of 1-24.5 years.

**Table 1:** Clinical characteristics of GATA2-deficient patients.

Pt	Age at presentation/Age at diagnosis/Delay of diagnosis (years)	Gender/ethnicity	Consanguinity/ Family history	*GATA2* variant^‡^	Clinical presentation^+++^							Treatment	Outcome/ Current age (years)/ Follow up period (years)
					Infectious				Hematological	GI	Pulmonary		
					Viral	Bacterial	Fungal	Myco- bacterial					
P1	10.5/ 35/24.5	F/J	None/ None	c.1081C>T, p.Arg361Cys, exon 5/6 (missense)**^+^**	Refractory verruca vulgaris; Recurrent CMV infections.	Recurrent sinusitis; Severe *Pseudomon as* cellulitis;	Onychomycosis	None	Cytopenia; MDS	Chronic diarrhea	None	None	Alive/ 39/28.5
P2	26.5/ 28/1.5	M/A	None/None	c.988C>T, Arg330*, exon 4/6 stop-gain (nonsense)**^++^**	None	*Moraxella catarrhalis* sepsis	None	None	Cytopenia; Necrotizing lymphadenitis	None	Lung fibrosis	Ceftriaxone and amikacin	Deceased/ 1.6
P3	23/ 24/1	M/A	None/None	c.177C>A, p.Tyr59*, exon 2/6 stop-gain (nonsense), novel	None	Neutropeni c fever	None	*MAC* BM infection	Cytopenia, HSM; Lymphadenopathy	Choleostatic liver disease	Bilateral pulmonary consolidations, Paraseptal pulmonary emphysema	Isoniazid , rifampicin, pyrazinamide, and ethambutol; Prednisone 40 mg/day	Alive/ 27/4
P4	36/ 47/11	F/J	None/None	c.610dup, p.Arg204Profs*78, exon 3/6 (frameshift), novel	None	*Bartonella* Lung abscess; recurrent pneumonia; *Acinetobact er* infection.	None	None	Cytopenia; MDS	None	Pulmonary alveolar proteinosis	Ceftriaxone Minocycline Coliracin	Alive/ 53/17

Pt, Patient; F, Female; M, Male; A , Arab; J, Jew; CMV, Cytomegalovirus; MAC, Mycobacterium Avium complex; GI, Gastrointestinal; BM, Bone marrow; HSM, hepatosplenomegaly; MDS, Myelodysplastic syndrome; **^‡^** All Gata2 variants are haploinsufficiency;**^+^** GATA2 variant was previously reported by Hsu et al. (3) and Svobodova et al. (7); **^++^** GATA2 variant was previously reported by West (9), Pasquet (6) and Zhang (2) et al. ^+++^None of the patients had malignant manifestations.

**Figure 1:**
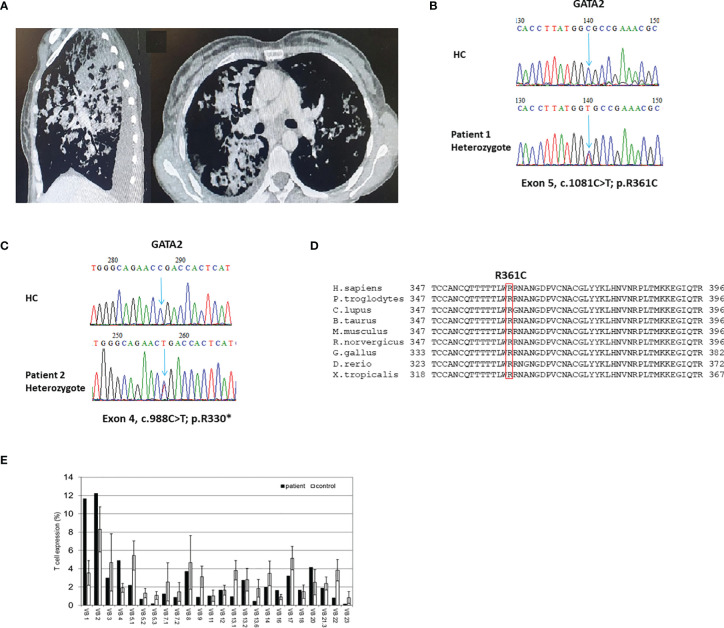
Clinical, genetic, and immune characteristics of GATA2 deficiency. **(A)** Sagittal and axial images of chest computed tomography scan of patient (P)1. Noted are bilateral consolidations constituting a radiological picture compatible with pulmonary alveolar proteinosis;** (B, C)** Sanger sequencing validation of the *GATA2* heterozygote variants found in exome sequencing analyses of P1 **(B)** and P2 **(C)**. HC–Healthy Control. **(D)** Multiple sequence alignment of the GATA2 amino acid region containing the conserved human mutated Arginine 361 residue (boxed in red) of P1 across different species. H.sapiens-Human; P.troglodytes, Chimpanzee; C.lupus, ;Wolf; B.taurus, Cattle; M.musculus, Mouse; R.norvergicus, Rat; G.gallus, Chicken; D.rerio, Zebrafish; X.tropicalis, Frog. **(E)** T-cell receptor v-β repertoire flow cytometry analysis of P1. A polyclonal repertoire can be seen with clonal expansions of vβ1 and vβ2.

There was notable phenotypic variability (**Table 1**). Hematological presentations consisted of cytopenia in all patients and MDS in two patients (P1 and P4). Malignancy was noted in none of the patients. All four patients had infectious manifestations, including recurrent viral (P1: CMV and HPV), non-tuberculous mycobacterial (P2), fungal (P1), and serious bacterial infections (SBI: P1-P4). SBI consisted of *Pseudomonas* spp. cellulitis (P1), *Moraxella catarrhalis* sepsis (P2), neutropenic fever (P3), and *Bartonella* spp. lung abscess with *Acinetobacter* spp. sepsis (P4). Pulmonary manifestations included lung fibrosis, paraseptal emphysema, and PAP (**Figure 1A**) in P2, P3, and P4, respectively.

### Genetic Diagnosis

ES was used to diagnose P1-P3. Representative Sanger sequencing chromatograms of P1 and P2 are presented in **Figures 1B, C**, respectively. P4 was diagnosed by Sanger sequencing of all coding exons in GATA2. Identified variants in GATA2 consisted of missense (P1), nonsense (P2 and P3), and small duplication (P4). Variants were found in the ZF-1 (P2), ZF-2 (P1), and non ZF (P3, P4) regions of GATA2. Previously reported GATA2 deficiency variants were identified in P1 (3, 7) and P2 (2, 6, 16). Two patients, P3 and P4, were found to harbor novel loss-of-function variants in GATA2 (c.177C>A; p.Tyr59* and c.610dup; p.Arg204Profs*78, respectively). The disease related ZF-2 missense variant observed in P1 is located in a highly conserved area of the gene (**Figure 1D**). Conservation status of P1 is of interest due to a missense variant within the ZF-2 domain, in contrast to the other 3 *GATA2* variants that induce premature stop codons and are therefore demonstrative of loss-of-function variants. All variants in our cohort were predicted to be pathogenic. ES in P1-P3 revealed no co-variants in other genes.

### Hematological Workup

The hematological workup is presented in **Table 2**. Peripheral blood cell counts revealed thrombocytopenia in P2-P4. Anemia was found in all patients (hemoglobin range: 8.8-11.1 g/dL; normal range: 13.5-17.5 g/dL). White blood cell counts yielded leukopenia in P1-P3; all three patients had lymphopenia with absolute lymphocyte counts ranging from 109 to 660 cells x 10^9^/L (1100-3800 x 10^9^/L). Monocytopenia was noted in all four patients, with a range of 0-30 (200-1000) x 10^9^/L. Neutropenia was observed in P2 and P3.

**Table 2:** Hematologic workup of patients with GATA2 deficiency.

Parameter		P1 (35 years)	P2 (28 years)	P3 (24 years)	P4 (47 years)	Normal range
Complete blood count	Absolute leukocyte count (10^9^/L)	4400	*3800*	*910*	5640	4000-10080
	Absolute lymphocyte count (10^9^/L)	*660*	*270*	*109*	1400	1100-3800
	Absolute monocyte count (10^9^/L)	*30*	*10*	*0*	*20*	200-1000
	Absolute neutrophil count (10^9^/L)	3670	*100*	*720*	4040	1800-7700
	Hemoglobin (gr/dL)	*11.1*	*9.0*	*9.2*	*8.8*	13.5-17.5
	Thrombocyte count (10^9^/L)	178	*8*	*27*	*118*	130-440
Bone marrow biopsy	Cytogenetics	Monosomy 7	NA	Normal	Normal	–
	Histopathology	Hypocellular; MDS	NA	Hematopoietic maturation arrest; Non-cesating granulomas with MAC; Increased amounts of macrophages and histyocytes	MDS	

NA, Data is not available; MDS, Myelodysplastic syndrome; MAC, Mycobacterium Avium complex. Values in italics are below reference range.

Bone marrow biopsy results were available in three patients (P1, P3, and P4) and revealed findings compatible with MDS in P1 and P4 and non-caseating granulomas in P3. Cytogenetic abnormalities were also found in P1 (monosomy 7).

### Immune Characteristics

The immune workup is detailed in **Table 3**. Lymphocyte subset phenotyping was available for P1-P3 (**Table 3**). Inverted CD4 to CD8 ratios were noted in P1-P3. P3 had reduced absolute numbers of NK cells, as well as reduced counts of CD3^+^, CD4^+^ and CD8^+^ lymphocytes. B cell lymphopenia was observed in all tested patients. Interestingly, although B-cell lymphopenia was observed in P1-P3, none of the patients had hypogammaglobulinemia (total immunoglobulin (Ig)G range: 1200-1650, normal range: 639-1349 mg/dL). Specific IgG titers were available for three patients (P1, P3, and P4). IgG titers were positive for Varicella-Zoster virus, rubella, and CMV (P1 and P4), as well as hepatitis A virus (P3 and P4).

**Table 3:** Immune characteristics of patients with GATA2 deficiency.

Parameter			P1 (35 years)	P2 (28 years)	P3 (24 years)	P4 (47 years)	Normal range^++^
Absolute leukocyte count (cells/mm^3^)			4670	3440	*330*	5640	4000-10080
Absolute lymphocyte count (cells/mm^3^)			1605	1926	*109*	1400	959-3644
Peripheral blood lymphocyte subsets (cells/mm^3^)	T cells	CD3^+^	1476	1772	*107*	NA	700-2508
		CD4^+^	883	867	*17*	NA	464-1721
		CD8^+^	818	809	*69*	NA	135-852
		CD4/CD8 ratio	*1.08*	*1.07*	*0.25*	NA	1.50-3.50
	NK cells	CD56^+^	**995**	308	*55*	NA	82-594
	B cells	CD20^+^	*64*	*39*	*0*	NA	92-515
TCR v-β repertoire			Normal/ polyclonal Clonal expansion of vβ1 and vβ2	NA	NA	NA	–
Serum immunoglobulins		IgG (mg/dL)	1200	1220	**1530**	**1650**	639-1349
		IgA (mg/dL)	110	159	182	NA	70-312
		IgM (mg/dL)	125	118	*27*	204	56-352
Specific IgG antibodies+		VZV (AI)	**Pos**	NA	NA	**Pos**	0-1.1
		Rubella (IU/mL)	**Pos**	NA	NA	**400**	0-30
		HBV surface (mU/mL)	*Neg*	NA	*Neg*	*Neg*	0-0.05
		HAV (S/CO)	NA	NA	**11.400**	**11.410**	0
		EBV EBNA (U/mL)	NA	*Neg*	NA	NA	0-20
		CMV (AU/mL)	**Pos**	*Neg*	NA	**Pos**	0-14
		Isohemaglutinins (IgM)	**Anti A (1:128) Anti B (1:128)**	NA	NA	NA	–

NA, Data is not available; NK, Natural killer cells; TCR, T-cell Receptor; TREC, T-cell receptor excision circles; PHA, Phytohemagglutinin; HC, Healthy Control; Ig, Immunoglobulin; VZV, Varicella Zoster virus; HBV, Hepatitis B virus; HAV, Hepatitis A virus; EBV, Epstein-Barr virus; EBNA, Epstein-Barr nuclear antigen; CMV, Cytomegalovirus; Pos, Positive; Neg, Negative. +Numerical values are presented when available. **^++^** Reference range for adult lymphocyte subsets were taken from Apoil et al. (15). Values in bold in italics are above and below reference range, respectively.

TCR v-β repertoire were analyzed in one patient. Flow cytometric analysis revealed a polyclonal TCR v-β repertoire, though clonal expansion of vβ1 and vβ2 was noted (**Figure 1E**).

### Treatment and Outcome

Patients were treated with antibiotics and anti-mycobacterials (**Table 1**). Follow-up period from initial presentation ranged between 1.6-28.5 years. None of the patients underwent hematopoietic stem cell transplantation (HSCT). Of the four patients, one died from overwhelming sepsis (P2) and three are currently alive (current age: 27-53 years).

## Discussion

In this report, we describe our experience with GATA2 deficiency manifesting in adult life. Of the four patients in our cohort, three presented with symptoms in adulthood with no relevant clinical course during childhood. In these patients, the first clinical manifestation of GATA2 deficiency occurred in the second and third decades of life. Thus, the diagnosis delay was up to 24.5 years.

GATA2 haploinsufficiency was previously reported to have incomplete penetrance, with reports of first manifestation of mycobacterial infections at a mean age of 22.5 years (16). Thus, understanding the impact of aging on GATA2-deficient HSPCs is critical. GATA2 is a master transcription factor involved in HSPC maintenance and proliferation (17). *GATA2*-knockout mice are born healthy, though defects in hematopoiesis can be found from birth (3, 17). Aging GATA2-deficient HSPCs are characterized by enhanced apoptosis and decreasing numbers (17). Therefore, GATA2 deficiency constitutes a unique primary immune deficiency (PID), as it can first manifest in adult life upon an age-related decline in the number of GATA2-deficient HSPCs (5).

In addition to the varying age of presentation, GATA2 deficiency is also characterized by its broad phenotypic spectrum. Increased susceptibility to infections is the most common manifestation of GATA2 deficiency (3) and was evident in our cohort, occurring in all four patients due to viral, bacterial, and mycobacterial pathogens. Notably, P1 presented with persistent verruca vulgaris and monocytopenia was the diagnostic clue suggestive of GATA2 deficiency.

Other known hematological and non-hematological manifestations, such as monosomy 7, MDS, and PAP (3), were also observed in our patients, further emphasizing the vast phenotypic diversity of GATA2 deficiency (3). This phenotypic diversity was also evident in the severity of infections. While P1 was managed without prophylactic antibiotics and had low rate of infections, P2 died from overwhelming sepsis.

Thus, delayed diagnosis, late onset in adult life, and clinical diversity are key factors that make the management of GATA2-deficient patients difficult. Two of the patients in our cohort were found to harbor novel *GATA2* variants, and both presented with symptoms and were diagnosed in adult life, further emphasizing the challenges for physicians when treating these patients.

The laboratory and clinical features of our cohort correspond well with the current literature. B- cell lymphopenia, as well as monocytopenia, are found in more than 75% of GATA2-deficient patients (3). MDS is found in up to 84% of patients (5), including two of our patients. PAP, which is caused by impaired macrophage function, and paraseptal emphysema were both found in our cohort. These findings were previously reported in a large cohort focusing on pulmonary manifestations in GATA2-deficient patients (18).

None of our patients developed myeloid malignancies and none had undergone HSCT. Allogeneic HSCT is thought to be effective in the treatment of GATA2 deficiency and has been shown to reverse PAP with favorable outcomes (3, 19). However, recommending HSCT to GATA2-deficient patients is challenging due to the broad clinical spectrum, incomplete penetrance, poor genotype–phenotype correlation and the lack of predictive factors for progression to MDS or myeloid malignancies. Thus, there is a need to balance between reducing the risk for MDS and hematological malignancies and possible HSCT complications. Monosomy 7, as was the case in P1 of our cohort, has been related to worse MDS outcome (20). Fortunately, P1’s BM has recovered from monosomy 7 and had no signs of MDS or malignancy in repeated BM biopsies.

Reported allogeneic HSCT in GATA2-deficient patients is low (~ 35%) (21). However, in a recent retrospective study of 14 GATA2 deficiency patients in Norway, Jørgensen et al. reported that 79% of their GATA2 deficiency patients had undergone allogeneic HSCT, mainly due to a well-established genetic screening program (9). Jørgensen et al, recommended close monitoring, annual BM investigations and recommended that a history of disseminated viral infection, aggressive HPV infection (particular with dysplasia), or myeloid clonal disease should be considered as indications for allogeneic HSCT (9).

Our study has several limitations. The most important limitation is its size and retrospective design. However, we think it adds to the accumulating data and emphasizes disease characteristics, which are unique to this rare PID.

In conclusion, GATA2 deficiency constitutes a PID that can present in adult life. With a variable range of clinical manifestations and severity, diagnosis and management are difficult. Thus, a high index of suspicion and careful monitoring are needed when treating these patients.

## Data Availability Statement

The datasets presented in this article are not readily available because uploading and publication of exome sequencings have not been approved by the patients. Requests to access the datasets should be directed to the corresponding authors.

## Ethics Statement

The studies involving human participants were reviewed and approved by IRB of Hadassah and Sheba Medical Centers. Written informed consent for participation was not required for this study in accordance with the national legislation and the institutional requirements.

## Author Contributions

OS, conceptual design, data collection, and manuscript writing. NS, data collection and treatment of patients. AS, genetic consultation, figure design, and manuscript revisions. AL, immune workup. OB and NM, genetic workup. YT, manuscript revisions. MS, manuscript revisions and treatment of patients. RS, HY, and OT, treatment of patients, study supervision, and manuscript revisions. All authors contributed to the article and approved the submitted version.

## Conflict of Interest

The authors declare that the research was conducted in the absence of any commercial or financial relationships that could be construed as a potential conflict of interest.

## Publisher’s Note

All claims expressed in this article are solely those of the authors and do not necessarily represent those of their affiliated organizations, or those of the publisher, the editors and the reviewers. Any product that may be evaluated in this article, or claim that may be made by its manufacturer, is not guaranteed or endorsed by the publisher.
